# On-Demand Microwave-Assisted Fabrication of Gelatin Foams

**DOI:** 10.3390/molecules23051121

**Published:** 2018-05-09

**Authors:** Shane D. Frazier, Anastasia N. Aday, Wil V. Srubar

**Affiliations:** 1 Materials Science and Engineering Program, University of Colorado Boulder, Boulder, CO 80309, USA; shane.frazier@colorado.edu (S.D.F.); anastasia.aday@colorado.edu (A.N.A.); 2 Department of Civil, Environmental, and Architectural Engineering, University of Colorado Boulder, Boulder, CO 80309, USA

**Keywords:** gelatin, foam, microwave processing

## Abstract

Ultraporous gelatin foams (porosity >94%, ρ ≈ 0.039–0.056 g/cm^3^) have been fabricated via microwave radiation. The resulting foam structures are unique with regard to pore morphology (i.e., closed-cell) and exhibit 100% macroporosity (pore size 332 to 1700 μm), presence of an external skin, and densities similar to aerogels. Results indicate that the primary foaming mechanism is governed by the vaporization of water that is tightly bound in secondary structures (i.e., helices, β-turns, β-sheets) that are present in dehydrated gelatin films but not present in the foams after microwave radiation (700 Watts).

## 1. Introduction

Foams have found application in drug delivery [1], catalyst supports [2], absorbents [3], and thermal insulation [4], among other areas [5]. Typically, foams are created from a variety of materials, including metals, such as aluminum [6], and polymers, including polystyrene and polyurethanes [7,8]. Foamed materials exhibit a wide range of densities (0.01 to 0.9 g/cm^3^) and porosities (0.4–99.9%) [9,10,11,12]. Biobased foams are a class of porous, lightweight materials that have been fabricated from abundant biopolymers, such as cellulose [13,14], alginate [15,16] and gelatin [17,18]. Biobased foams have the advantage of exhibiting material properties similar to the traditional foams, while being renewable and, often, biodegradable [19,20,21,22].

Gelatin is a biopolymer that has found many applications in the food [23], pharmaceutical [24], and biomedical industries [25]. Gelatin is derived from the collagen of various fish, bovine, and porcine species. One of the most abundant sources of gelatin is porcine skin, making up 46% of the worldwide production of gelatin in 2007 [26]. Porcine gelatin is obtained from the acid hydrolysis of collagen, where the primary amino acid composition consists of glycine, proline, and hydroxyproline in various abundancies [27]. Gelatin can be used to create hydrogels via a sol-gel transition. Upon cooling of the sol, the amino acid residues allow for the partial reformation of triple helices into secondary helix structures, which are considered the driving force behind the sol-gel transition of gelatin [28].

Porous gelatin foams have been created using a variety of fabrication methods, with freeze-drying (or modified versions) being the most widely employed [18,29,30,31,32,33]. In this method, the water in gelatin solutions is frozen, then subsequently lyophilized, yielding an open pore structure. Freeze-drying methods have been used to create porous structures in other biopolymers, including chitosan [34] and silk fibroin [35]. Gelatin-based foams have also been created using a modified gas foaming method [36], an evaporation-based method [22], a combined freeze-drying and salt-leaching technique [37], electrospinning [38], and 3-D printing [39].

This study presents a novel method to fabricate gelatin-based foams with ultra-macroporosity using microwave radiation. The method did not necessitate the utilization of solvents (other than water), freeze-drying, gases, high temperatures, or high pressures. Instead, the method presented herein utilizes microwave energy to vaporize water that is tightly bound within dehydrated gelatin hydrogel films. The purpose of this study was to fully elucidate the fundamental mechanism governing the foaming process and to characterize the resulting gelatin foams that were fabricated using this method.

## 2. Results

### 2.1. Gelatin Foam and Pore Morphology

**Figure 1:**
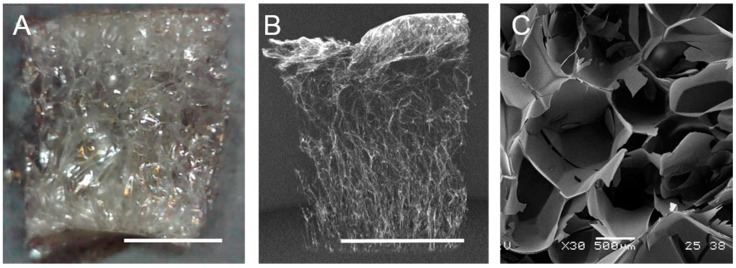
(**A**) Bulk foam sample, (**B**) MXCT 3D foam reconstruction, (**C**) SEM images of foam cross-section. Scale bar in (**A**), (**B**) is 5 mm. Scale bar in (**C**) is 500 μm.

**Figure 2:**
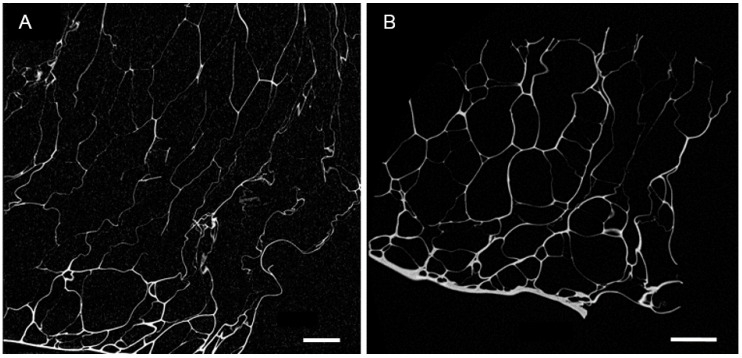
(**A**) 2D MXCT image of pore morphology in direction perpendicular to skin and (**B**) 2D MXCT images of pore morphology in direction parallel to skin. Scale bar is 1 mm.

Representative images of (1) bulk gelatin foams, (2) 3D reconstruction of the foam, and (3) scanning electron microscope (SEM) micrograph of the foam cross-section are shown in Figure 1. The resulting foams exhibit external skins that were primarily smooth, with an average thickness of 14 μm. The internal foam structure, which displays birefringence (Figure 1A), is comprised of irregularly shaped closed-cell pores with minimal interconnectivity. The bulk density of pores is higher (and pore diameters smaller) near the skins (775 ± 224 μm pore size near skin). Figure 2 provides 2D Micro X-ray Computed Tomography (MXCT) images of pore morphology perpendicular to and parallel to the skins. Table 1 summarizes the density, porosity, pore size, skin and edge thickness. Figure 2 and the pore size data in Table 1 show that the pores are marginally larger in dimension in the direction perpendicular to the external skins.

### 2.2. Helical Structure of Gelatin Powder, Films, and Foam Structures

**Figure 3:**
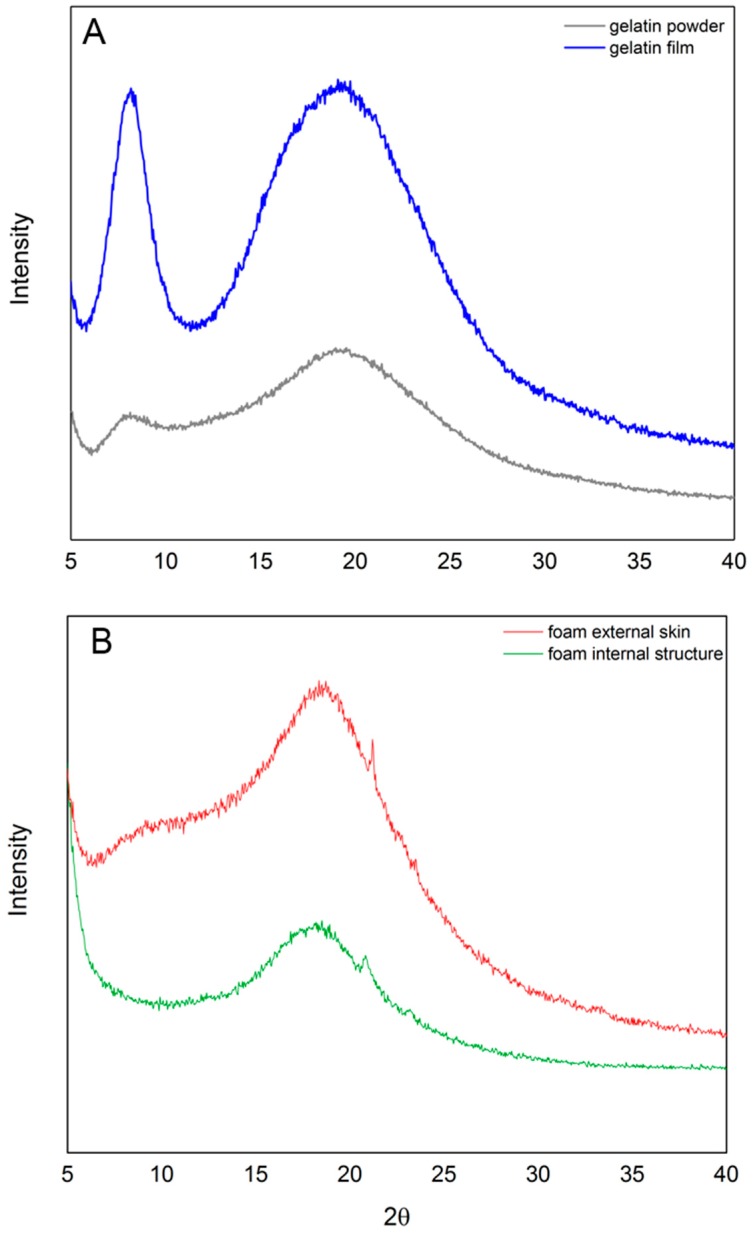
Representative XRD diffractograms of (**A**) dehydrated gelatin powder and dehydrated gelatin film and (**B**) foam external skin and foam internal structure.

The X-ray diffractograms in Figure 3 show the relative helical content of gelatin powder, gelatin films, and gelatin foams produced using the microwave-assisted method. The characteristic peak at 2θ ≈ 8° corresponds to the repeat distance (1.1 nm) between each turn in the helical structure of gelatin [40]. The area of this peak has been used previously as a measure of relative helix content [22,28,40,41]. Accordingly, relative helix contents of 10.2, 60.5, 0, and 0 were found for the gelatin powder, gelatin films, foam external skin, and foam internal structure, respectively. The broad peak centered at 2θ ≈ 19° for all samples is distinctive of gelatin and has been identified as the spacing between polypeptide chains. The microwave-processed foams display an additional peak at 2θ ≈ 21°.

### 2.3. Water Content Analysis

#### 2.3.1. Thermogravimetric Analysis

**Figure 4:**
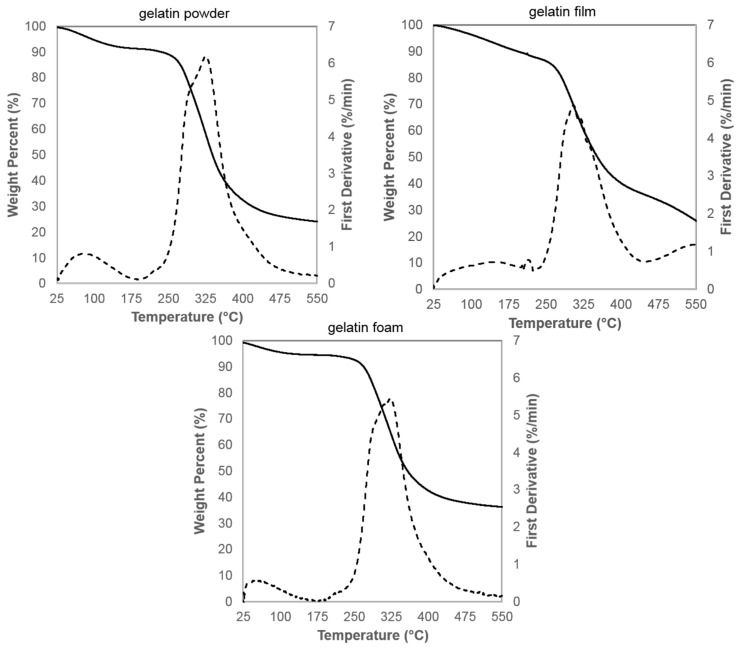
TGA traces (—) with accompanying first derivate (---), reported in wt %/min, for the gelatin powder, film, and foam.

The thermogravimetric analysis (TGA) traces with accompanying first derivative (DTG) curves are shown in Figure 4 for gelatin powder, dehydrated gelatin films, and resulting gelatin foams. The powder and foam undergo a change in weight percent from 25 °C to 150 °C due to free water loss [22,42] and up to 250 °C that corresponds to the loss of loosely bound water [22,43,44]. From Figure 4, the gelatin foam contains the lowest water content (5%), followed by gelatin powder (8%), and gelatin films (12%) as expected. The second event between 250 °C and 440 °C, where major weight loss is observed in all samples, can be attributed to the degradation of peptide bonds [22,42,44]. The TGA curves for the gelatin film displayed a small peak at approximately 210 °C that corresponds to the thermal unfolding of gelatin [45]. The shapes of the TGA curves and the onset of degradation at approximately 250 °C for gelatin powder, films, and foams suggest that the microwave processing method used to foam the dehydrated gelatin films causes little to no thermal degradation of gelatin.

#### 2.3.2. Differential Scanning Calorimetry

**Figure 5:**
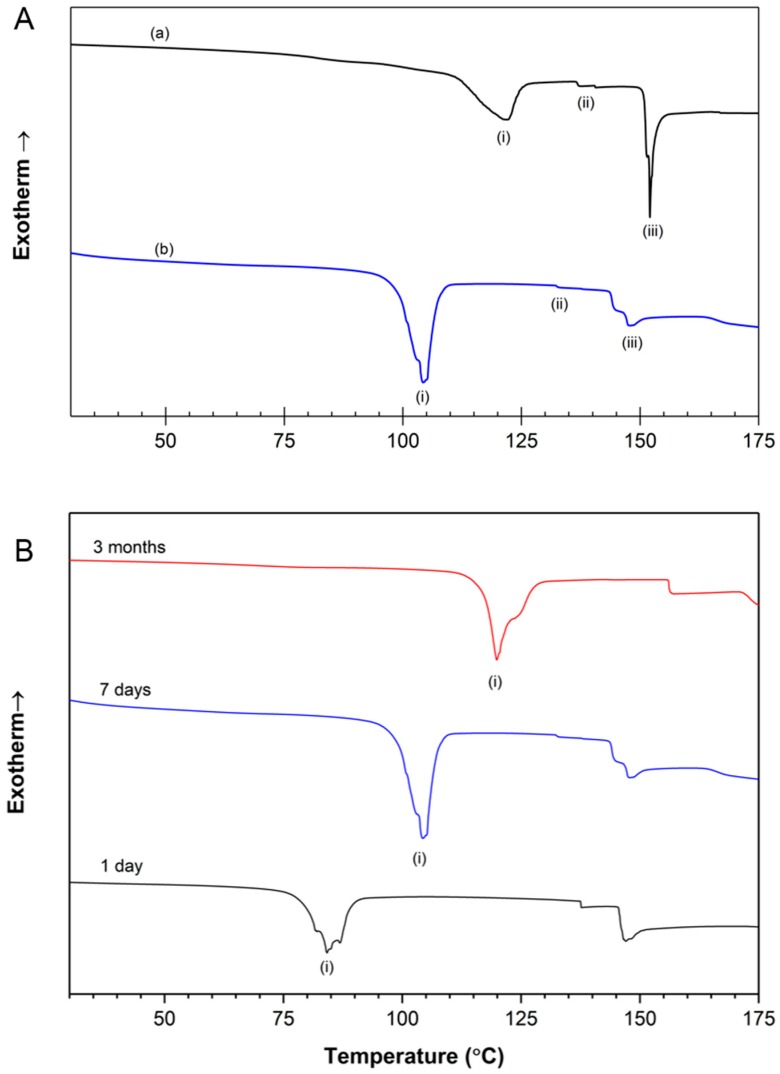
(**A**) DSC thermogram of gelatin film showing vaporization of (i) loosely and (iii) tightly bound water in (a) gelatin powder and (b) gelatin film. (**B**) DSC thermogram of gelatin films at 1 day, 7 days, and 3 months of dehydrating (i.e., aging).

The differential scanning calorimetry (DSC) thermograms for gelatin powder and dehydrated film are shown in Figure 5A. As previously reported [46], gelatin films do not exhibit a melting feature at the commonly reported melting temperature of 30–50 °C [47,48] after 3 days of dehydration (i.e., aging). Upon cooling and second heating, no thermal events were observed (data not shown). Upon first heating, the first peak centered at approximately 105 °C, labeled (i) in Figure 5A, can be attributed to the vaporization (evaporation) of loosely bound water.

The enthalpy of vaporization for loosely bound water, peak (i) in Figure 5A, was calculated for the unmodified gelatin powder and the dehydrated gelatin film (Table 2). The enthalpy of vaporization for the film matches closely with previous research [22]. The peak temperatures for the enthalpy of vaporization, defined herein as the temperature at the peak (i) minima for all samples is also listed in Table 2.

In Figure 5A, the thermal feature labeled (ii) occurs at approximately 135 °C and appears similar to (but is likely not) a glass transition. Feature (ii) is likely a relaxation event corresponding to the initial vibration of tightly bound water that induces chain mobility within the gelatin framework. Feature (ii) is not seen on cooling or second heating; thus, this feature is likely not a glass transition. The event with an onset temperature between 145 and 150 °C, labeled (iii) in Figure 5A, corresponds with the vaporization of tightly bound water and the onset of foaming. Peak (iii), or what is characterized in this work as the foaming peak, was highly irregular for all samples due to changes in volume and corresponding changes in the surface area that was in contact with the aluminum pan. The peak has aspects of both a first- and second-order phase transition. Therefore, enthalpy of this peak was difficult to determine and thus is not reported herein. However, the onset temperature for peak (iii) is reported in Table 2. In Figure 5B, the dehydrated gelatin films were compared, showing loosely bound water peaks (i) shifting from ~84 °C for 1-day-aged films to approximately ~120 °C for films as old as 3 months.

## 3. Discussion

Water is required for formation and stabilization of helices in gelatin [40,49], and several types of water, including tightly and loosely bound water, exist within the resulting hydrogel [22]. While water may be tightly bound at charged amino acid residues and both inside and outside of helical structures, loosely bound water refers to the polymolecular layers of water adsorbed to the sample surface [22]. Helical structures, identified by the XRD peak at 2θ ≈ 8° in Figure 3, that are initially present in the raw gelatin powders and dehydrated gelatin films are not present in the foam skin or internal foam structure. The XRD data in Figure 3 support the prevailing theory that vaporization of tightly bound water is the principle mechanism for microwave-assisted fabrication of gelatin foams.

Literature reports the large, broad peak centered at approximately 2θ ≈ 19° in Figure 3 as the spacing between polypeptide chains [50,51] with a range between 2θ ≈ 19° and 22° [22,28,40,50]. The variable range is likely due to variation in amount of random coil, helical structure, and other secondary structures that may be present in gelatin. In Figure 3, this peak is shifted in the microwaved gelatin samples to 2θ ≈ 18° which corresponds to the loss of helical structure and is indicative of a higher random-coil content. The microwave-fabricated foams exhibit an additional peak at 2θ ≈ 21° that is present in neither film nor powder. Given that (1) this peak is relatively sharp, (2) foams exhibit birefringence (Figure 1A), and (3) this peak falls within the range attributed to polypeptide spacing in gelatin, we posit that stress-induced alignment of polypeptide chains is occurring during the foaming process.

Thermal images, shown in Figure S1 in the supplementary information, show that foaming is induced when samples reach a temperature of approximately 150 °C. These data, in conjunction with the shapes of the TGA curves (Figure 4) and the onset of degradation at approximately 250 °C for gelatin powder, films, and foams, suggest that the microwave-assisted foaming process causes little to no thermal degradation of gelatin. The two distinct weight loss event and thermal stability observed in TGA data align well with previous research [22,42,44].

The TGA and DTG curves (Figure 4) for gelatin films consistently displayed an event at ~210 °C. The gelatin films were powderized for TGA analysis, and therefore have a much higher surface area to volume ratio than the bulk films. The authors anticipated that the increased surface area would inhibit foaming because the evaporating water in the powder would not be able to build as high of an internal pressure. Therefore, the event is likely not attributable to foaming, but rather to thermal unfolding of gelatin. Previous research reported a peak centered at ~215 °C in DSC thermograms for bovine gelatin with low water concentrations [45]. The thermal unfolding likely caused the sample to expand inducing movement in the TGA sample holder, which would be characteristic of the shape and size of the event in the TGA curve.

The DSC thermograms (Figure 5) display two distinctive peaks for the vaporization of loosely (~95 °C) and tightly (~150 °C) bound water in gelatin films. This finding agrees with previously reported results in gelatin hydrogel literature, where endothermic peaks centered at approximately 95 °C were attributed to the volatilization and/or evaporation of water [42,52]. The gelatin powder has a higher peak value for (i), ~120 °C, which is likely due to polymolecular water, a form of loosely bound water [22,40], evaporating over time. This evaporation is evidenced in DSC thermogram (B) (Figure 5) showing the loosely bound peak (i) of films shift to ~120 °C as they dehydrate (i.e., age) for a longer period of time.

While vaporization of tightly bound water has been previously leveraged to fabricate gelatin foams, radiative heat transfer of the microwave-assisted method imparts unique morphologies and properties to gelatin foams. Previous research has utilized thermal energy to induce foaming of dehydrated gelatin films [22] by placing the films in an oven at 150 °C. When samples are placed in an oven, heat transfer occurs primarily through a combination of conduction and convection and creates a thermal gradient between the surface and internal material of the film. Contrastingly, radiative heat transfer occurs when emitted microwaves are absorbed by the sample. Microwave radiation is absorbed by the electrically dipolar water molecules due to polarization induced by the external oscillating electric field [53]. We propose that the difference in overall pore morphology between gelatin films exposed to thermal energy (aligned tubular pore structures) versus microwave energy is due to the underlying phenomenon in how the microwave energy is interacting with tightly bound water.

Microwave processing is rapid and efficient, which could be advantageous with regard to scalability in comparison to the conventional methods of foam production. Other studies [54,55,56] report on the microwave processing of starch-based foams, but do not develop densities as low as the method presented herein (densities are >0.1 g/cm^3^). Water content is an important factor in the foaming process, as it acts like a blowing agent and aids in the formation of the foam morphology. As in the method presented herein, water interacts with the microwave radiation causing water to vaporize or evaporate inducing pore structure formation.

The microwave method presented herein produces a gelatin foam material with pore sizes of 332 to 1700 μm which are comparably larger than other fabrication methods. The freeze-drying method (or a modified version of it) generally results in pore sizes of 100 to 700 μm in gelatin-based foams [17,18,31], while less common techniques, such as thermally induced foaming [22], gas foaming [36], and salt leaching [37], result in pore sizes of 320–367 μm, 280–550 μm, and 250–420 μm, respectively. All aforementioned methods result in a combination of open- and closed-cell pore morphologies with the exception of the thermally induced method [22]. The porosities obtained herein (~95%) are similar to the highest porosities reported in studies utilizing all other methods previously employed. 

## 4. Materials and Methods

### 4.1. Gelatin Film Preparation

Porcine gelatin (PG) of BioReagent grade was obtained from Sigma Aldrich in powder form. Ultrapure water obtained from a Millipore purification system (Milli-Q) was first heated to 40 °C. PG was added and manually stirred until dissolved. Upon dissolution at 40 °C, stirring was continued for 10 additional minutes. Solutions were cast into plastic 52 ± 3 mm diameter petri dishes and allowed to dehydrate at ambient conditions for at least six days to form dehydrated films.

### 4.2. Foam Fabrication

Dehydrated gelatin films aged in ambient conditions were foamed using a microwave oven (Dansby) at a wattage of 700 W and a frequency of 2450 MHz for 30 to 50 s. The resulting foams were used to study helical (i.e., secondary structure) content, density, pore size, and morphology.

### 4.3. X-ray Diffraction

X-ray diffraction (XRD) patterns were collected using a Bruker D2 Phaser Benchtop XRD equipped with a Cu radiation source (λ = 1.54184 Å) operating at 30 kV and 10 mA. Data were recorded in the range of 2θ = 5° to 40° at a scanning rate 1°/min. Unexpanded aged (dried) films, interior foam pore structure, and foam skin samples were powderized using liquid nitrogen and subsequently placed on a Si, P-type B-doped, zero diffraction plate obtained from MTI Corporation with a small amount of Vaseline (X-Alliance, Hamburg, Germany). Gelatin powder was tested as received. All diffraction patterns were analyzed using DIFFRAC.SUITE EVA version 3.0 (Bruker, Billerica, MA, USA).

### 4.4. Thermogravimetric Analysis

Thermogravimetric analysis (TGA) of gelatin powder, aged (dried) films, and foams was performed using a TA Instruments Q50 TGA. Gelatin powder was tested as received. Gelatin films were aged (dried) for at least six days prior to testing. Gelatin foams were produced following the procedure outlined in Section 4.2. All samples were tested under N2 gas. Samples were first equilibrated at 25 °C followed by heating at a rate of 10 °C/min to 625 °C. All weight-percent traces were differentiated with respect to time in TA Universal Analysis software.

### 4.5. Differential Scanning Calorimetry

Thermal data for samples aged (dried) for at least six days were collected using a TA Instruments Q2000 Differential Scanning Calorimeter (DSC) in a N_2_ environment using a purge rate of 50 mL/min. Samples of dehydrated gelatin films were placed in hermetically sealed aluminum pans. The samples were first equilibrated to 15 °C followed by heating at a rate of 10 °C/min to 180 °C. The samples were then cooled at a rate of 10 °C/min to −10 °C and then heated again to 180 °C at a rate of 10 °C/min. Enthalpy calculations and transition temperatures were analyzed using Universal Analysis software (TA Instruments, New Castle, DE, USA).

### 4.6. Thermal Imaging

Thermal images were captured with a FLIR i3 Infrared Camera. Gelatin films were exposed to microwave energy (700 Watts), and images were captured at 10, 20, and 30 s to determine the temperature corresponding to onset of foaming.

### 4.7. Density and Porosity Measurements of Foams

The density of foams and films were measured using the ratio of mass to volume. The average and standard deviation of measured densities of 10 samples was reported. The volumes of foam samples were determined by cutting samples into rectangular shapes and measuring dimensions with calipers. The volume of films samples was directly measured using calipers. Masses were determined using an analytical balance (Mettler Toledo, XS105DU, Columbus, OH, USA). Bulk porosity of the resulting foams was determined using the following equation [57].

Porosity (%) = (V_b_ − V_g_)/Vb × 100 = (V_b_ − (W_b_/ρ))/V_b_ × 100

where V_b_ is the volume of the foam (cm^3^), V_g_ is the volume of gelatin only (cm^3^), W_b_ is the total ambient-condition mass of the foam (g), and ρ is the density of gelatin (1.037 g/cm^3^) [57].

### 4.8. Characterization of Foam Morphology

Scanning electron microscopy (SEM, Hitachi SU3500, Tokyo, Japan) was performed using an accelerating voltage of 10 kV to analyze pore morphology and pore size. Fractured samples were attached to aluminum stubs using carbon tape and then sputter-coated with Pt (Cressington 108auto) for 30–35 s at 40 mA (~4.0–4.3 nm) in an Ar-rich environment.

In addition, foams were analyzed using Micro X-ray Computed Tomography (MXCT, ZEISS Xradia 520 Versa, Thornwood, NY, USA). The source voltage was set to 30 or 40 kV. The source current, source location, detector location and exposure time were varied to obtain desired resolution (voxel size). Pore size was determined by manually drawing a line across a minimum of 100 pores on SEM and 2D MXCT images in Image J 1.48v software (National Institutes of Health, Bethesda, MD, USA) and Dragonfly 3.1 software (Object Research Systems, Montreal, QC, Canada). Due to the pore shape, pore size was recorded as the largest pore diameter in the directions perpendicular and parallel to the skins. 3D reconstructions were made using Dragonfly 3.1 software.

## 5. Conclusions

A rapid fabrication method that leverages microwave-induced vaporization of tightly bound water to create gelatin foams was presented in this work. Data from gelatin samples substantiate that the resulting foams are distinct in pore size and shape from those fabricated using conventional preparation methods (i.e., freeze-drying, gas foaming, salt leaching). The foaming mechanism (i.e., vaporization of tightly bound water) was elucidated using a combination of differential scanning calorimetry (DSC), X-ray diffraction (XRD), and an analysis of water content (TGA) in gelatin films before and after foaming. The DSC thermograms of dehydrated gelatin films exhibited a characteristic peak centered at ~150 °C, which can be attributed to the vaporization of tightly bound water in the protein structures. Secondary (helical) structures were identified using XRD in the dehydrated gelatin films but were not present in the foam samples, substantiating that the foaming mechanism is primarily governed by the evaporation of water that is tightly bound in dehydrated gelatin films.

## Figures and Tables

**Table 1:** Density, porosity, and pore sizes of porcine (PG) gelatin foams prepared via microwave-based method.

Property	Value
Density (g/cm^3^)	0.039–0.056
Average porosity (%)	95 ± 1
Pore size perpendicular to skin (μm)	1337 ± 260
Pore size parallel to skin (μm)	1037 ± 289
Skin thickness (μm)	14 ± 8
Edge thickness (μm)	10 ± 5

**Table 2:** Summary of DSC data collected from dehydrated porcine gelatin (PG) powder and gelatin film.

Sample	Average Enthalpy (J/g) of Loosely Bound Water	Average Peak Temperature (°C) of Loosely Bound Water	Average Onset Temperature (°C) of Tightly Bound Water
PG powder	11.90	118.2	151.2
PG film	34.83	104.3	147.6

## Supplementary Materials

The following are available online, Figure S1: Thermal images of film samples after (**1**) 10 s, (**2**) 20 s, (**3**) 30 s of exposure to microwave energy.

## References

[1] Hegge A.B., Anderson T., Melvik J.E., Kristensen S., Tønnesen H.H. (2010). Evaluation of Novel Algniate Foams as Drug Delivery Systems in Antimicrobial Photodynamic Therapy (aPDT) of Infected Wounds—An In Vitro Study: Studies on Curcumin and Curcuminoides XL. J. Pharm. Sci..

[2] Thompson C.R., Marin P., Díez F.V., Ordóñez S. (2013). Evaluation of the use of ceramic foams as catalyst supports for reverse-flow combustors. Chem. Eng. J..

[3] Yang Y., Deng Y., Tong Z., Wang C. (2014). Multifunctional foams derived from poly(melamine formaldehyde) as recyclable oil absorbents. J. Mater. Chem. A.

[4] Basso M.C., Li X., Fierro V., Pizzi A., Giovando S., Celzard A. (2011). Green, formaldehyde-free, foams for thermal insulation. Adv. Mater. Lett..

[5] Hu W., Chen R., Xie W., Zou L., Qin N., Bao D. (2014). CoNi_2_S_4_ Nanosheet Arrays Supported on Nickel Foams with Ultrahigh Capacitance for Aqueous Asymmetric Supercapacitor Applications. ACS Appl. Mater. Interfaces.

[6] Nieh T.G., Higashi K., Wadsworth J. (2000). Effect of cell morphology on the compressive properties of open-cell aluminum foams. Mater. Sci. Eng. A.

[7] Han X., Zeng C., Lee L.J., Koelling K.W., Tomasko D.L. (2003). Extrusion of polystyrene nanocomposite foams with supercritical CO_2_. Polym. Eng. Sci..

[8] Kuhn J., Ebert H.P., Arduini-Schuster M.C., Büttner D., Fricke J. (1992). Thermal transport in polystyrene and polyurethane foam insulations. Int. J. Heat Mass Transf..

[9] Hanssen A.G., Enstock L., Langseth M. (2002). Close-range blast loading of aluminum foam panels. Int. J. Impact Eng..

[10] Blaker J.J., Maquet V., Jérôme R., Boccaccini A.R., Nazhat S.N. (2005). Mechanical properties of highly porous PDLLA/Bioglass^®^ composite foams as scaffolds for bone tissue engineering. Acta Biomater..

[11] Sepulveda P., Ortega F.S., Innocentini M.D.M., Pandolfelli V.C. (2004). Properties of Highly Porous Hydroxyapatite Obtained by the Gelcasting of Foams. J. Am. Ceram. Soc..

[12] Jiang B., He C., Zhao N., Nash P., Shi C., Wang Z. (2015). Ultralight metal foams. Sci. Rep..

[13] Sehaqui H., Salajková M., Zhou Q., Berglund L.A. (2010). Mechanical performance tailoring of tough ultra-high porosity foams prepared from cellulose I nanofiber suspensions. Soft Matter.

[14] Dash R., Li Y., Ragauskas A.J. (2012). Cellulose nanowhisker foams by freeze casting. Carbohydr. Polym..

[15] Vincent T., Dumarzert L., Dufourg L., Cucherat C., Sonnier R., Guibal E. (2017). New alginate foams: Box-Behnken design of their manufacturing; fire retardant and thermal insulating properties. J. Appl. Polym. Sci..

[16] Chen H., Wang Y., Sánchez-Soto M., Schiraldi D.A. (2012). Low flammability, foam-like materials based on ammonium alginate and sodium montmorillonite clay. Polymer.

[17] Panzavolta S., Torricelli P., Casolari S., Parrilli A., Amadori S., Fini M., Bigi A. (2017). Gelatin Porous Scaffolds as Delivery Systems of Calcium Alendronate. Macromol. Biosci..

[18] Amadori S., Torricelli P., Panzavolta S., Parrilli A., Fini M., Bigi A. (2015). Highly Porous Gelatin Reinforced 3D Scaffolds for Articular Cartilage Regeneration. Macromol. Biosci..

[19] Salgado P.R., Schmidt V.C., Molina Ortiz S.E., Mauri A.N., Laurindo J.B. (2008). Biodegradable foams based on cassava starch, sunflower proteins and cellulose fibers obtained by a baking process. J. Food Eng..

[20] Wang H.J., Rong M.Z., Zhang M.Q., Hu J., Chen H.W., Czigány T. (2008). Biodegradable Foam Plastics Based on Castor Oil. Biomacromolecules.

[21] Mahmood N., Yuan Z., Schmidt J., Xu C. (2015). Preparation of bio-based rigid polyurethane foam using hydrolytically depolymerized Kraft lignin via direct replacement or oxypropylation. Eur. Polym. J..

[22] Frazier S.D., Srubar W.V. (2016). Evaporation-based method for preparing gelatin foams with aligned tubular pore structures. Mater. Sci. Eng. C.

[23] Etxabide A., Uranga J., Guerrero P., de la Caba K. (2017). Development of active gelatin films by means of valorisation of food processing waste: A review. Food Hydrocoll..

[24] García-González C.A., Jin M., Gerth J., Alvarez-Lorenzo C., Smirnova I. (2015). Polysaccharide-based aerogel microspheres for oral drug delivery. Carbohydr. Polym..

[25] Su K., Wang C. (2015). Recent advances in the use of gelatin in biomedical research. Biotechnol. Lett..

[26] Gómez-Guillén M.C., Pérez-Mateos M., Gómez-Estaca J., López-Caballero E., Giménez B., Montero P. (2009). Fish gelatin: A renewable material for developing active biodegradable films. Trends Food Sci. Technol..

[27] Hafidz R., Yaakob C. (2011). Chemical and functional properties of bovine and porcine skin gelatin. Int. Food Res. J..

[28] Gioffrè M., Torricelli P., Panzavolta S., Rubini K., Bigi A. (2012). Role of pH on stability and mechanical properties of gelatin films. J. Bioact. Compat. Polym..

[29] Zhang F., He C., Cao L., Feng W., Wang H., Mo X., Wang J. (2011). Fabrication of gelatin-hyaluronic acid hybrid scaffolds with tunable porous structures for soft tissue engineering. Int. J. Biol. Macromol..

[30] Kang H., Tabata Y., Ikada Y. (1999). Fabrication of porous gelatin scaffolds for tissue engineering. Biomaterials.

[31] Ren L., Tsuru K., Hayakawa S., Osaka A. (2002). Novel approach to fabricate porous gelatin-siloxane hybrids for bone tissue engineering. Biomaterials.

[32] Islam M.M., Khan M.A., Rahman M.M. (2015). Preparation of gelatin based porous biocomposite for bone tissue engineering and evaluation of gamma irradiation effect on its properties. Mater. Sci. Eng. C.

[33] Gentile P., Mattioli-Belmonte M., Chiono V., Ferretti C., Baino F., Tonda-Turo C., Vitale-Brovarone C., Pashkuleva I., Reis R.L., Ciardelli G. (2012). Bioactive glass polymer composite scaffolds mimicking bone tissue. J. Biomed. Mater. Res. A..

[34] Ma J., Wang H., He B., Chen J. (2001). A preliminary in vitro study on the fabrication and tissue engineering applications of a novel chitosan bilayer material as a scaffold of human neofetal dermal fibroblasts. Biomaterials.

[35] Nazarov R., Jin H., Kaplan D.L. (2004). Porous 3-D scaffolds from regenerated silk fibroin. Biomacromolecules.

[36] Poursamar S.A., Hatami J., Lehner A.N., Silva C.L., Ferreira F.C., Antunes A.P.M. (2015). Gelatin porous scaffolds fabricated using a modified gas foaming technique: Characterisation and cytotoxicity assessment. Mater. Sci. Eng. C.

[37] Liu X., Ma P.X. (2009). Phase separation, pore structure, and properties of nanofibrous gelatin scaffolds. Biomaterials.

[38] Powell H.M., Boyce S.T. (2008). Fiber density of electrospun gelatin scaffolds regulates morphogenesis of dermal-epidermal skin substitutes. J. Biomed. Mater. Res. A.

[39] An J., Teoh J.E.M., Suntornnond R., Chua C.K. (2015). Design and 3D Printing of Scaffolds and Tissues. Engineering.

[40] Yakimets I., Wellner N., Smith A.C., Wilson R.H., Farhat I., Mitchell J. (2005). Mechanical properties with respect to water content of gelatin films in glassy state. Polymer.

[41] Bigi A., Panzavolta S., Rubini K. (2004). Relationship between triple-helix content and mechanical properties of gelatin films. Biomaterials.

[42] Peña C., de la Caba K., Eceiza A., Ruseckaite R., Mondragon I. (2010). Enhancing water repellence and mechanical properties of gelatin films by tannin addition. Bioresour. Technol..

[43] Schiraldi D.A., Gross R.A., Cheng H.N., Smith P.B. (2015). Green Polymer Aerogels. Green Polymer Chemistry: Biobased Materials and Biocatalysis.

[44] Barreto P.L.M., Pires A.T.N., Soldi V. (2003). Thermal degradation of edible films based on milk proteins and gelatin in inert atmosphere. Polym. Degrad. Stab..

[45] Rahman M.S., Al-Saidi G., Guizani N., Abdullah A. (2010). Development of state diagram of bovine gelatin by measuring thermal characteristics using differential scanning calorimetry (DSC) and cooling curve method. Thermochim. Acta.

[46] Dorr D.N., Frazier S.D., Hess K.M., Traeger L.S., Srubar W.V. (2015). Bond strength of biodegradable gelatin-based wood adhesives. J. Renew. Mater..

[47] Gomez-Guillen M.C., Gimenez B., Lopez-Caballero M.E., Montero M.P. (2011). Functional and bioactive properties of collagen and gelatin from alternative sources: A review. Food Hydrocoll..

[48] Tseretely G.I., Smirnova O.I. (1992). DSC study of melting and glass transition in gelatins. J. Therm. Anal..

[49] Djabourov M., Leblond J., Papon P., Djabourov M., Leblond J., Papon P. (1988). Gelation of aqueous gelatin solutions. I. Structural investigation. J. Phys..

[50] Panzavolta S., Gioffrè M., Focarete M.L., Gualandi C., Foroni L., Bigi A. (2011). Electrospun gelatin nanofibers: Optimization of genipin cross-linking to preserve fiber morphology after exposure to water. Acta Biomater..

[51] Okuyama K. (2008). Revisiting the molecular structure of collagen. Connect. Tissue Res..

[52] Apostolov A.A., Fakirov S., Vassileva E., Patil R.D., Mark J.E. (1999). DSC and TGA studies of the behavior of water in native and crosslinked gelatin. J. Appl. Polym. Sci..

[53] Vollmer M. (2003). Physics of the microwave oven. Phys. Educ..

[54] Zhou J., Song J., Parker R. (2006). Structure and properties of starch-based foams prepared by microwave heating from extruded pellets. Carbohydr. Polym..

[55] Lopez-Gil A., Silva-Bellucci F., Velasco D., Ardanuy M., Rodriguez-Perez M.A. (2015). Cellular structure and mechanical properties of starch-based foamed blocks reinforced with natural fibers and produced by microwave heating. Ind. Crops Prod..

[56] Torres F.G., Boccaccini A.R., Troncoso O.P. (2006). Microwave Processing of Starch-Based Porous Structures for Tissue Engineering Scaffolds. J. Appl. Polym. Sci..

[57] Wu X., Liu Y., Wen P., Zhang Y., Long Y., Wang X., Guo Y., Xing F., Gao J. (2010). Preparation of aligned porous gelatin scaffolds by unidirectional freeze-drying method. Acta Biomater..

