# Supplementary material for: On-Demand Microwave-Assisted Fabrication of Gelatin Foams
Source: Molecules. 2018 May 9;23(5):1121. doi: 10.3390/molecules23051121 (PMC6100080; doi:10.3390/molecules23051121)
Supplement: Supplementary file 1 [file molecules-23-01121-s001.pdf]

molecules

On-Demand Microwave-Assisted Fabrication of Gelatin Foams

Shane D. Frazier [1], Anastasia N. Aday [1] and Wil V. Srubar III [1,2,*]

1 Materials Science and Engineering Program, University of Colorado Boulder, Boulder, CO 80309, USA; shane.frazier@colorado.edu (S.D.F.); anastasia.aday@colorado.edu (A.N.A.)
2 Department of Civil, Environmental, and Architectural Engineering, University of Colorado Boulder, Boulder, CO80309-0428, USA
* Correspondence: wsrubar@colorado.edu; Tel.: +1-303-492-2621

Supplementary Information

Thermal images of a dehydrated film sample as it undergoes microwave heating are shown in Figure S1. The panels show (from left to right) that the film experiences heating where the onset of foaming is observed at 150 °C after approximately 30 seconds. Additionally, a change in cross-sectional area of the film in panel (3) indicates onset of foaming.

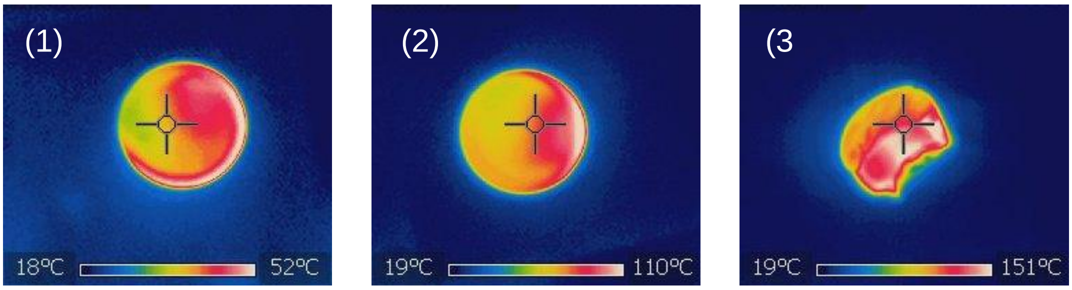

Figure S1. Thermal images of film samples after (1) 10 s, (2) 20 s, (3) 30 s of exposure to microwave energy.
